# New insights into total and resting energy expenditure using state-of-the-art methods in cancer survivors: a cross-sectional study

**DOI:** 10.1016/j.ajcnut.2025.09.032

**Published:** 2025-09-26

**Authors:** Ana Paula Pagano, João Felipe Mota, Sarah A Purcell, Iasmin M Sousa, Hongyi Cai, Richard JE Skipworth, Tom Preston, Pierre Singer, Michael B Sawyer, Rajavel Elango, Peter J Walter, Carla M Prado

**Affiliations:** 1 Department of Agricultural, Food, & Nutritional Science, University of Alberta, Edmonton, Alberta, Canada; 2 School of Nutrition, Federal University of Goiás, Goiás, Brazil; 3 Department of Medicine, Southern Medical Program, Centre for Chronic Disease Prevention and Management, University of British Columbia, Kelowna, British Columbia, Canada; 4 School of Health and Exercise Sciences, University of British Columbia—Okanagan, Kelowna, British Columbia, Canada; 5 Health Sciences Center, Department of Health Sciences, Federal University of Rio Grande do Norte, Natal, Rio Grande do Norte, Brazil; 6 Clinical Mass Spectrometry Core Laboratory, National Institute of Diabetes and Digestive and Kidney Diseases, National Institutes of Health, Bethesda, Maryland, United States; 7 Clinical Surgery, University of Edinburgh, Edinburgh, United Kingdom; 8 Stable Isotope Biochemistry Laboratory, Scottish Universities Environmental Research Centre, University of Glasgow, Glasgow, United Kingdom; 9 Nutrition Research, Rabin Medical Center, Beilinson Hospital, Petah Tikva, and Lab on Computerized Decision Making, Dina Recanati School of Medicine, Reichman University, Herzliya, Israel; 10 Department of Oncology, University of Alberta, Edmonton, Alberta, Canada; 11 Department of Pediatrics, University of British Columbia, Vancouver, British Columbia, Canada; 12 BC Children’s Hospital Research Institute, BC Children’s Hospital, Vancouver, British Columbia, Canada; 13 School of Population and Public Health, Faculty of Medicine, University of British Columbia, Vancouver, British Columbia, Canada

**Keywords:** colorectal cancer, energy metabolism, energy expenditure, resting energy expenditure, total energy expenditure, predictive equations, doubly labeled water, whole-room indirect calorimeter, body composition

## Abstract

**Background:**

Precise measurement of energy expenditure is essential for guiding nutritional care after cancer treatment. However, commonly used predictive equations may be inaccurate for individuals recovering from cancer. Leveraging state-of-the-art methods can offer valuable insights into the actual energy requirements of cancer survivors upon treatment completion.

**Objectives:**

The aim of this study was to characterize total (TEE) and resting energy expenditure (REE) and to assess the accuracy of predictive equations against measured values in posttreatment colorectal cancer survivors (CRCSs).

**Methods:**

In this cross-sectional study, 4 TEE and 22 REE equations were compared against doubly labeled water and whole-room indirect calorimeter, respectively. Accuracy was assessed via paired *t* test, Bland-Altman analysis, and the proportion of predictions within 10% of measured values. Pearson correlations investigated relationships between percentage bias and variables of interest [e.g., age, weight, BMI (in kg/m^2^), and body composition].

**Results:**

Twenty participants (equal sex distribution; mean ± SD age: 61.4 ± 14.1 y; BMI: 28.8 ± 6.4) were included. Most had a history of colon cancer (55%) and stage III disease (75%). Predictive equations (predicted TEE range: ∼2060–2500 kcal/d; predicted REE range: ∼1230–1730 kcal/d) commonly underestimated measured TEE (∼2460 ± 680 kcal/d) (*n* = 2, 50%) and REE (∼1700 ± 330 kcal/d) (*n* = 19, 86.4%). Dietary Reference Intake equations with estimated physical activity level had the highest individual-level accuracy for TEE prediction but still resulted in substantial intra-individual variability (∼≤1400 kcal error). BMI and body composition were positively related to percentage bias in TEE but not REE equations. For REE, the Johnstone and Harris-Benedict equations showed the best individual-level agreement but still exhibited high intraindividual variability, with errors up to ∼≤460 kcal and ∼≤530 kcal, respectively.

**Conclusions:**

The majority of CRCSs exhibit higher energy expenditure than estimated by standard prediction equations, underscoring the need to validate these equations in populations with cancer to optimize accuracy. Improved methods for assessing energy expenditure are needed to guide long-term survivorship care.

## Introduction

Energy expenditure in patients with cancer may be influenced by tumor activity, inflammation, hormonal alterations, and changes in body composition [1]. A sustained negative energy balance—where energy intake remains lower than total energy expenditure (TEE)—can lead to weight loss and adversely affect prognosis [[2], [3], [4]]. Conversely, sustained positive energy balance can result in weight gain and an increased risk of obesity, which may also be detrimental [5].

Characterizing energy balance after cancer treatment is particularly important given the growing number of cancer survivors and the complex, multifaceted factors that may impact their energy expenditure. For example, colorectal cancer (CRC) survivors (CRCS) remain at high risk of obesity and other comorbidities after active treatment because of body composition changes, lasting treatment side effects, psychosocial factors, and nutritional challenges, among others [5,6]. Malnutrition, also prevalent in this population [7], can persist beyond treatment completion [8]. Some individuals may also seek support to achieve a desired body weight—whether through weight loss or gain—further highlighting the need for accurate assessment of energy requirements in this population.

Evidence regarding the optimal method for determining TEE and its primary component, resting energy expenditure (REE), in clinical settings remains inconclusive. Although reference methods provide accurate assessments of energy requirements, their use in clinical practice is limited by cost, need for trained personnel, and time required for assessments, among other barriers [9]. Thus, predictive equations are commonly used to estimate energy requirements, despite evidence of their inaccuracy in patients with cancer [[10], [11], [12]]. Even less is known about their accuracy in individuals who have survived cancer. Notably, current energy intake recommendations for both patients with cancer and survivors assume that TEE is similar to that of healthy individuals, ranging from 25 to 30 kcal/kg body weight per day [13]. However, this one-size-fits-all approach may be inadequate for those who have completed cancer treatment, given their unique metabolic alterations and increased vulnerability to malnutrition, changes in body composition, and chronic disease.

To date, no studies have characterized TEE and REE using state-of-the-art methods in individuals who have survived cancer, nor have they evaluated the accuracy of commonly used clinical estimates against reference methods in this population. Characterizing their energy requirements is essential for guiding nutritional care after cancer treatment, as targeted strategies can reduce risk of secondary comorbidities (e.g., obesity), address treatment-related sequelae that may have impaired nutritional status, and ultimately improve outcomes [5]. Therefore, the objective of this study was to characterize, for the first time to our knowledge, TEE and REE in posttreatment CRCSs by comparing measured and estimated values using state-of-the-art methods. We used doubly labeled water (DLW) and a whole-room indirect calorimeter (WRIC), which are recognized as gold standard techniques for measuring TEE and REE, respectively [14,15]. We also evaluated, for the first time to our knowledge, accuracy of commonly used predictive equations in this population. We hypothesized that energy requirements would vary among CRCSs and that predictive equations would be inaccurate compared with reference methods, highlighting the need for personalized approaches in survivorship care.

## Methods

### Study design and population

This study aligns with our ongoing research interest in exploring energy expenditure in patients with cancer [16]. It is distinct from our previously published study [16], as the present study involved a separate cohort of individuals who had completed treatment for CRC. A checklist of items recommended for reporting cross-sectional studies, based on the STROBE statement [17], is available in Supplemental Table 1. Individuals who had completed chemotherapy for stage II or III CRC within the past 3 y, regardless of radiotherapy eligibility, were recruited from the Cross Cancer Institute, Edmonton, Alberta, Canada. Study assessments were completed at the Human Nutrition Research Unit (HNRU) at the University of Alberta between November 2019 and August 2022, and DLW analyses were completed in April 2023. We excluded individuals who had finished anticancer therapy or invasive surgery within the past month, had severe mobility issues (e.g., confined to a wheelchair), were taking medications that could affect body composition or energy metabolism (e.g., corticosteroids and hormone replacement), had a pacemaker, or were pregnant or lactating. We received ethics approval from the Health Research Ethics Board of Alberta (protocol number: HREBA.CC-15-0204), and all participants provided written informed consent before assessments.

### Pretest protocol

Participants fasted overnight (only water consumption) and were instructed to avoid alcohol and strenuous exercise for 24 h before the appointment. They were also instructed to arrive at the HNRU by car and take the elevator. Pretest protocols were verbally confirmed upon arrival, and participants were asked to complete a study-specific form that gathered information on demographics and diagnosis.

### Anthropometrics and body composition

Height and weight were measured with a Health-O-Meter Professional digital scale with height rod (model number: 597KL) after removal of shoes and heavy clothes. BMI was calculated according to the equation, weight in kilograms divided by height in square meters, and was categorized according to WHO classification: underweight, ≤18.5 kg/m^2^; normal weight, 18.6–24.9 kg/m^2^; overweight, 25–29.9 kg/m^2^; and obesity, ≥30 kg/m^2^ [18].

Body composition was assessed by dual-energy x-ray absorptiometry (DXA; Lunar iDXA, GE Healthcare; Encore 2001 software version 13.60) (median: 14 d apart from study visit date; range: 5–21 d). Fat mass (FM), fat-free mass (FFM), and appendicular lean soft tissue (ALST), which represents the sum of lean soft tissue from limbs, were reported and adjusted for height in square meters to express FM index (FMI), FFM index (FFMI), and ALST index (ALSTI), respectively. FM and FFM were also reported as a ratio (FM:FFM) to represent metabolic load and capacity, as explained elsewhere [19]. ALST was further normalized by weight and multiplied by 100 to express it as a percentage, as this approach is more recommended to identify sarcopenic obesity [20]. Body composition terminology is defined according to published standards [21].

### Measured TEE

TEE was measured with DLW over 14 d. Details on the study protocol, including isotope concentrations, dosing procedures, sample collections (before and after dose administration), analyses of isotope enrichments, and TEE calculations, have been described elsewhere [16]. Briefly, DLW isotope concentrations were prepared with 10 atom% oxygen-18 (^18^O) and 99.9 atom% deuterium (^2^H) based on 1 g ^18^O and 0.1 g ^2^H per kilogram of body weight. A single urine sample was collected before dosing participants, followed by multiple collections after dosing (4.5 and 6 h after dose administration, and 1–2 sample collections on the following days until day 14 after dose administration). Isotope enrichment from urine samples from before dose administration, 4.5 h and 6 h after administration, and days 3, 7, and 14 were analyzed at the Clinical Mass Spectrometry Core Laboratory, National Institute of Diabetes and Digestive and Kidney Diseases, at the National Institutes of Health. Isotope ratios were measured on a Thermo Delta V Advantage isotope ratio mass spectrometer with HDevice and GasBench peripherals. Each analytical batch included a water blank and low-enrichment and high-enrichment quality control (QC) samples, and the calibration range together with house blank/low/high QC samples extended beyond the range of enrichments observed in study specimens. HDevice measurements were performed in triplicate and GasBench measurements in duplicate. All participant samples fell within the instrument’s calibrated range; therefore, no values were below the limits of detection/quantification, and no censoring or substitution rules were required. The simplified equation from the International Atomic Energy Agency [22] and the Weir equation [23] were used to calculate carbon dioxide and TEE, respectively. The food quotient was assumed to be 0.86, representative of a typical diet on a population level [24]. QC measures included assessing ^18^O enrichment/intercept, linear fit of ^2^H and ^18^O slopes, elimination rates, dilution spaces, and residuals of predicted and measured isotopes, as previously described [16].

### Predicted TEE

In the absence of specific guidelines for cancer survivors, we applied the commonly recommended range of 25–30 kcal/kg to predict TEE. This range, originally developed for healthy subjects, is also reflected in cancer-specific guidelines [13]. Additionally, we calculated TEE using the recently updated Dietary Reference Intake (DRI) equations, incorporating each participant’s age, sex group, and physical activity level (PAL; described below) [25].

### Measured REE

REE was assessed using a WRIC. Participants lay on their back and rested without significant movement or falling asleep during the 1-h test. The first 30 min were discounted for acclimatization. REE was obtained by averaging the subsequent 30 min. The equipment was calibrated every morning before assessments as per the manufacturer’s recommendations. Volume of oxygen consumed and volume of carbon dioxide produced were measured with the Oxymat 6 O_2_ analyzer (Siemens AG) and the Advance Optima AO2000 Series CO_2_ analyzer (ABB Automation GmbH). During the test, differences in gas concentrations were calculated each minute by the Advance Optima AO2000 Series CO_2_ analyzer (ABB Automation GmbH) and the Oxymat 6 O_2_ analyzer (Siemens AG). Data were transferred from the gas analyzers to a computer using the National Instruments NI USB-6221 device (National Instruments Corporation) and PMCSS Software version 1.8 (Pennington Metabolic Chamber Software Suite, Pennington Biomedical Research Center). The abbreviated Weir equation was used to calculate REE [23].

### Predicted REE

Twenty-two equations were used to predict REE (Supplemental Table 2). Of those, 11 did not account for body composition, 10 included body composition data, and 1 was an aggregate REE of all equations. The aggregate REE was expressed as the average of each predictive equation herein, and it was compared against measured REE as previously done in another study by our group [11]. Equations were selected based on their frequency of use in clinical and research settings, as well as prior application in REE validation studies involving populations with cancer [11]. Actual body weight was used in all equations, as the use of adjusted body weight can introduce errors, particularly in individuals with obesity [26]. Injury factors were not applied, as this approach has been shown to overestimate REE in ambulatory patients [27]. Equations expressed in megajoules or kilojoules were converted to kilocalories for consistency and comparison.

### Physical activity level

Measured PAL was calculated as TEE divided by REE. To enhance clinical translation, PAL was estimated using the International Physical Activity Questionnaire (IPAQ) categories (i.e., 1 = low, 2 = moderate, and 3 = high), classified according to the questionnaire guidelines [28]. The IPAQ category for each participant was then converted into estimated PAL categories for calculating energy requirements using DRIs. Specifically, participants with an IPAQ = 1 had a PAL equivalent to the inactive DRI equation, whereas an IPAQ = 2 was equivalent to the low active DRI equation, and an IPAQ = 3 was equivalent to the active DRI equation. Physical activity was further treated as equivalent to the “inactive” PAL category of DRI equation, as generally reflective of this population for comparison between approaches.

### Sample size calculation

Our sample size calculation was based on detecting a clinically meaningful 5% difference between predicted TEE (using the 30 kcal/kg estimate) and measured TEE (obtained via DLW). Assuming an average energy requirement of 2000 kcal/d, a difference of 100 kcal/d was deemed meaningful [29]. Using a paired *t* test framework and assuming a SD of 100 kcal/d for differences between predicted and DLW-measured TEE, a sample size of *n* = 20 provided 80% power to detect this difference at a 2-sided α = 0.05. This corresponds to a 95% confidence interval (CI) half-width of approximately 44 kcal/d for the mean paired difference, ensuring adequate precision around our estimate.

In addition, we considered a precision/equivalence-based approach. Using the two one-sided tests framework with an equivalence margin of ±100 kcal/d (our predefined 5% clinical threshold) and the same SD assumption, the required sample size to achieve 80% power was approximately *n* = 8. With our sample size (*n* = 20), the effective equivalence margin narrows to approximately ±56 kcal/d, and the corresponding 90% CI half-width is approximately 39 kcal/d, demonstrating that our study exceeds the precision required to establish agreement within the clinically meaningful ±100 kcal/d threshold.

### Statistical analysis

Paired *t* tests (or Wilcoxon signed-rank tests for nonnormally distributed data) were used to compare measured and predicted energy expenditure (TEE and REE). Independent samples *t* test (or Mann-Whitney U test for nonnormally distributed data) were used to compare body composition between males and females. Levene’s test investigated variances and independent sample *t*-test assessed group differences in percentage bias between sexes based on predicted compared with measured TEE and REE. We conducted these analyses to investigate whether energy expenditure and body composition differed between males and females and to determine whether sex-stratified analyses were warranted, because body composition varies by sex and may influence energy expenditure [30]. We also investigated whether treatment duration and the time elapsed from the end of treatment to the study visit influenced TEE or REE using independent samples *t* tests, as the data were normally distributed. Values were presented as mean, SD, median, IQR, percentage, and/or range, where appropriate. Values were rounded in the abstract and discussion sections for clarity but are presented in full in the results section. There were no missing data for any variable; thus, no imputation was performed.

We used different approaches to investigate the accuracy and agreement of equations compared with measured TEE and REE, each providing different insights. First, data were tested for normality with the Shapiro-Wilk test, and paired samples *t* tests were used to compare differences between predicted and measured TEE and REE. Next, the Bland-Altman approach was used to assess the agreement of the equations compared with measured TEE and REE [31]. The Bland-Altman analysis further provided insights regarding individual variability and systematic differences between predicted and measured TEE and REE. Bias was calculated as the average difference between measured and predicted values to determine group-level agreement, where positive bias indicates overprediction and negative bias indicates underprediction of TEE or REE. Limits of agreement were calculated as the mean bias ±1.96 SD of the bias and interpreted as indicators of individual-level agreement. To enhance interpretation, absolute limits of agreement were calculated as the absolute difference between the lower and upper limits. To account for differences in body size, bias and limits of agreement were also expressed as percentages of measured TEE or REE. Individual-level accuracy was further assessed by calculating the proportion of individuals whose predicted TEE or REE was within ±10% of measured values. Although a 5% threshold (∼100 kcal/d) was used for sample size to ensure adequate power to detect small group-level differences, a 10% threshold was applied when assessing individual-level agreement, consistent with commonly cited cutoffs in the energy expenditure prediction literature [29]. Predictions >110% were considered overestimations and <90% as underestimations.

**FIGURE 1:**
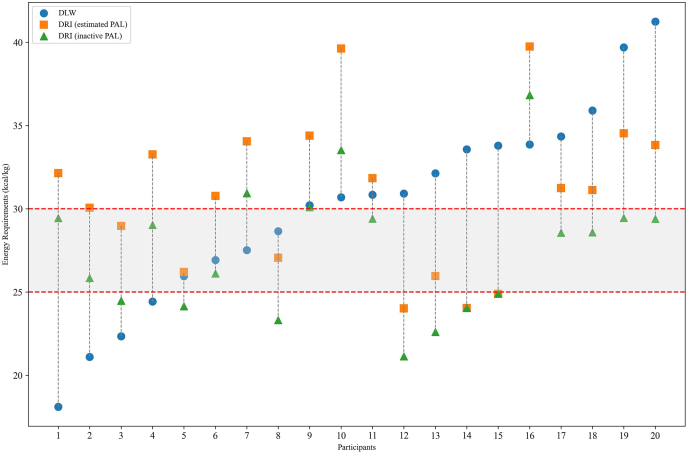
Each circle represents the total energy expenditure (TEE) by each survivor of colorectal cancer (*n* = 20) in this study, according to that measured by doubly labeled water (DLW). Each square represents the TEE estimated by the Dietary Reference Intake (DRI) equations using an estimated physical activity level (PAL) according to each participant’s age and sex. Each triangle represents the TEE estimated by the DRI with “inactive” PAL. Estimated PAL was estimated using the International Physical Activity Questionnaire categories converted into an estimated PAL according to DRI; inactive PAL refers to the physical activity equivalent to the “inactive” DRI equation. The box represents the normal range of intake recommended to healthy individuals (i.e., 25–30 kcal/kg body weight). The figure demonstrates that 80% (*n* = 16) of survivors of colorectal cancer do not have their total energy requirements met as measured by DLW according to that estimated by the 25–30 kcal/kg range. From those, 75% (*n* = 12) were underestimated and 25% (*n* = 4) overestimated. Compared with the DLW (within ±10% of measured TEE), the DRI (with estimated PAL) equations underestimated true energy requirements of *n* = 2 (10%) and overestimated of *n* = 7 (35%) participants, whereas the DRI (with inactive PAL) equations underestimated requirements of *n* = 9 (45%) and overestimated of *n* = 2 (10%) compared with the DLW method.

**FIGURE 2:**
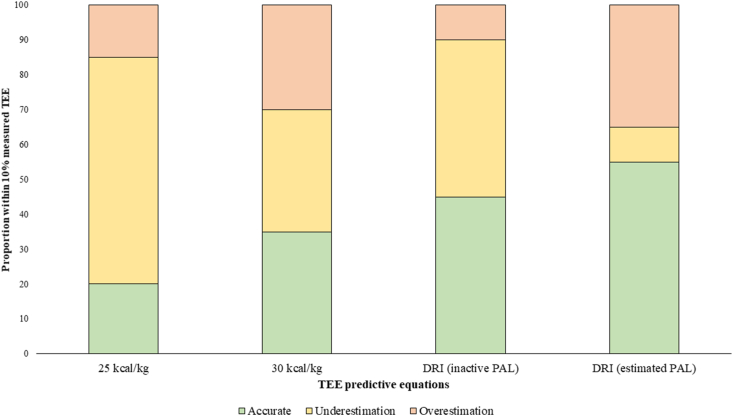
Proportion of total energy expenditure (TEE) predicted by 4 methods within 10% of that measured by doubly labeled water. Predictions <90% of the measured TEE were classified as underestimations, whereas predictions >110% were classified as overestimations. The most accurate equation was the Dietary Reference Intake (DRI) with estimated physical activity level (PAL), whereas the most inaccurate one was the 25 kcal/kg estimate. Normal range of intake recommended to healthy individuals is 25–30 kcal/kg. Estimated PAL was estimated using the International Physical Activity Questionnaire categories converted into their correspondent activity factors according to DRI. Inactive PAL refers to the physical activity equivalent to the “inactive” DRI equation.

**FIGURE 3:**
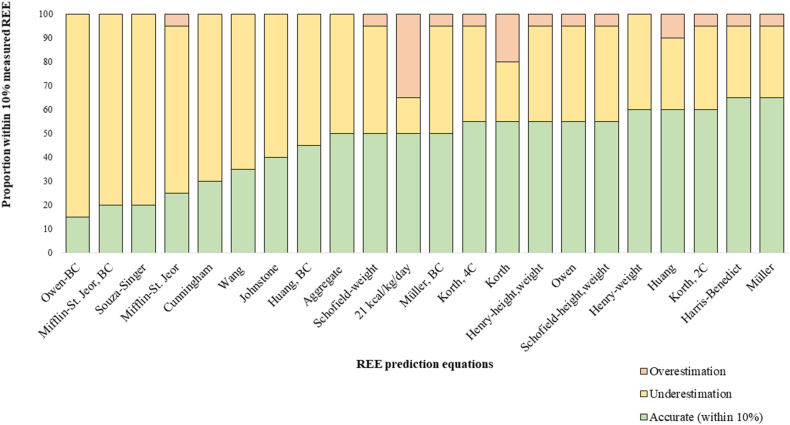
Proportion of resting energy expenditure (REE) predicted by 22 equations within 10% of that measured by a whole-room indirect calorimeter. Predictions <90% of the measured REE were classified as underestimations, whereas predictions >110% were classified as overestimations. The most accurate equations were Harris-Benedict and Müller (65% each), followed by Henry (weight only), Huang (weight only), and Korth [equation using 2-compartment model (2C)] (60% each) equations. Conversely, the most inaccurate ones were the Owen [body composition (BC)], followed by the Mifflin-St. Jeor (BC), and the Souza-Singer equations. 4C, equation using 4-compartment model; Aggregate = mean resting energy expenditure from all predictive equations (without and with body composition data).

**FIGURE 4:**
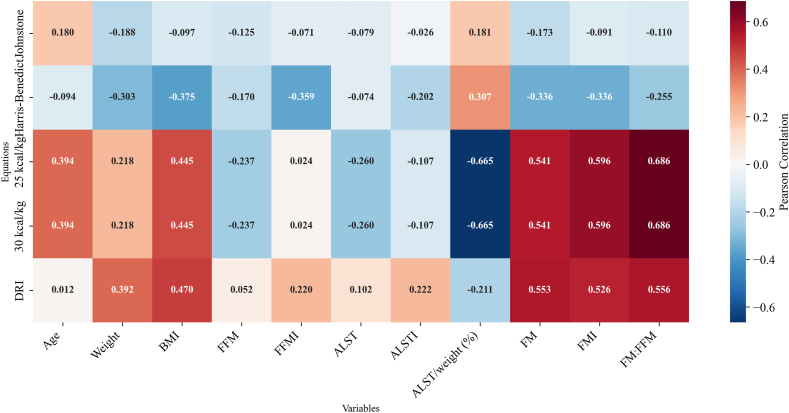
Heatmap of the correlation of percentage bias between total energy expenditure or resting energy expenditure equations with characteristics of 20 colorectal cancer survivors. Physical activity level was estimated with the International Physical Activity Questionnaire categories converted into their correspondent activity factors according to the Dietary Reference Intake (DRI). Normal range of intake recommended to healthy individuals is 25–30 kcal/kg. Appendicular lean soft tissue (ALST)/weight (%) represents ALST normalized by weight and multiplied by 100 to identify sarcopenic obesity. Fat mass (FM):fat-free mass (FFM) represents metabolic load and capacity. ALSTI, appendicular lean soft tissue index; FFMI, fat-free mass index; FMI, fat mass index.

Proportional bias was determined using Pearson correlation between bias and mean of measured and predicted TEE or REE. Pearson correlations were further used to investigate relationships between energy expenditure (and percentage bias) and participants’ characteristics, including age, weight, BMI, and body composition. Correlation strength was classified as very weak (*r* = 0–0.19), weak (*r* = 0.20–0.39), moderate (*r* = 0.40–0.59), strong (*r* = 0.60–0.79), and very strong (*r* = 0.80–1.0) [32]. Significance was considered when *P* < 0.05. Data were analyzed using JASP (version 0.17.1.0; JASP Team, 2025). FIGURE 1, FIGURE 4 were created with plotMakerPRO (https://www.plotmakerpro.com/), and FIGURE 2, FIGURE 3 were created with Microsoft Excel 365.

## Results

A total of 20 posttreatment CRCSs with equal sex distribution and stages II or III disease were enrolled. Supplemental Figure 1 illustrates the participant flowchart, and participant characteristics are presented in Table 1. The majority had a history of colon cancer and a stage III diagnosis. Most participants had obesity (*n* = 8, 40%), followed by overweight (*n* = 5, 25%), whereas 1 was classified as underweight (*n* = 1, 5%). Treatment included adjuvant chemotherapy (*n* = 12), or a combination of adjuvant chemotherapy with radiation (prechemotherapy, concurrent chemotherapy, and postchemotherapy, *n* = 1), or neoadjuvant radiation and chemotherapy followed by adjuvant chemotherapy (*n* = 7). The median time from treatment initiation to completion was 179 d (IQR: 122.5 d), and the median time from treatment completion to the study visit was 396 d (IQR: 635 d). Treatment duration and the time elapsed from the end of treatment to the study visit did not influence TEE or REE (data not shown).

**TABLE 1:** Characteristics of 20 survivors of colorectal cancer^1^.

Characteristic	Total (*N* = 20)	Males (*n* = 10)	Females (*n* = 10)
Age (y)	61.4 ± 14.1 (34–82)	61.6 ± 14.9 (35–82)	61.2 ± 14.0 (34–76)
Body weight (kg)	82.5 ± 17.3 (47.3–114.2)	88.1 ± 15.2 (65.1–108.2)	76.9 ± 18.2 (47.3–114.2)
Height (m)	1.7 ± 0.1	1.8 ± 0.1	1.6 ± 0.1
BMI (kg/m^2^)	28.8 ± 6.4 (17.9–43.6)	28.2 ± 4.4 (20.7–35.1)	29.3 ± 8.1 (17.9–43.6)
REE, WRIC (kcal/d)	1697 ± 331 (1182.0–2386.0)	1839 ± 344 (1386.0–2386.0)	1555.3 ± 263 (1182.0–2099.0)
REE, WRIC (kcal/kg)	20.8 ± 2.3	20.9 ± 1.6	20.7 ± 2.9
TEE, DLW (kcal/d)	2467 ± 684 (1462.0–4215.0)	2834 ± 679 (2167.0–4215.0)	2100 ± 477 (1462.0–2964.0)
TEE, DLW (kcal/kg)	30.1 ± 5.9	32.4 ± 6.0	27.8 ± 5.1
FFM (kg)	52.5 ± 10.4 (37.2–75.3)	59.2 ± 9.6 (49.0–75.3)	45.8 ± 6.0 (37.2–60.2)
FFMI (kg/m^2^)	18.1 ± 2.7 (14.1–23.0)	18.9 ± 2.4 (15.7–21.7)	17.3 ± 2.8 (14.1–23.0)
ALST (kg)	22.4 ± 5.3 (14.9–35.2)	26.2 ± 5.0 (20.0–35.2)	18.6 ± 2.5 (14.9–23.4)
ALSTI (kg/m^2^)	7.7 ± 1.4 (5.5–10.0)	8.3 ± 1.3 (6.6–10.0)	7.0 ± 1.1 (5.5–8.7)
FM (kg)	29.6 ± 11.0 (9.7–52.1)	28.7 ± 9.5 (14.3–44.3)	30.5 ± 12.8 (9.7–52.1)
FMI (kg/m^2^)	10.5 ± 4.5 (3.7–19.9)	9.2 ± 3.1 (4.2–14.4)	11.7 ± 5.4 (3.7–19.9)
IPAQ			
Category 1	2 (10)	1 (5)	1 (5)
Category 2	7 (35)	4 (20)	3 (15)
Category 3	11 (55)	5 (25)	6 (30)
Measured PAL	1.45 ± 0.23 (0.97–1.83)	1.54 ± 0.23 (1.10–1.83)	1.35 ± 0.18 (0.97–1.60)
Tumor type			
Colon	11 (55)	6 (60)	5 (50)
Rectal	9 (45)	4 (40)	5 (50)
Stage			
II	5 (25)	2 (20)	3 (30)
III	15 (75)	8 (80)	7 (70)

Abbreviations: ALST, appendicular lean soft tissue; ALSTI, appendicular lean soft tissue index; BMI, body mass index; DLW, doubly labeled water; FFM, fat-free mass; FFMI, fat-free mass index; FM, fat mass; FMI, fat mass index; IPAQ, International Physical Activity Questionnaire; PAL, physical activity level; REE, resting energy expenditure; SD, standard deviation; TEE, total energy expenditure; WRIC, whole-room indirect calorimeter.

1 Values are presented as mean ± SD, mean ± SD (range), or n (%). IPAQ categories 1, 2, and 3 were converted into an estimated physical activity level according to Dietary Reference Intake and corresponded to “inactive,” “low active,” and “active,” respectively. Measured PAL refers to the ratio between TEE and REE measured by DLW and WRIC, respectively.

Levene’s test indicated equal variances in percentage bias between sexes for both TEE and REE (*P* > 0.05), allowing for use of independent samples *t* tests to assess group differences. No significant differences in percentage bias between predicted and measured TEE or REE were found between sexes (data not shown). Because TEE equation biases and most REE equation biases did not differ significantly between sexes, no sex stratification was performed.

TEE estimated from 25 kcal/kg (2062 ± 433 kcal/d, *P* = 0.003) and TEE using the inactive PAL in the DRI equation (2232 ± 351 kcal/d, *P* = 0.036) underestimated measured TEE on a group level (2467 ± 684 kcal/d; range: 1462–4215 kcal/d) (Table 1). In contrast, TEE calculated as 30 kcal/kg (2475±520 kcal/d, *P* = 0.948) or using individual estimated PAL in the DRI (2498±410 kcal/d, *P* = 0.706) was not different than measured TEE on a group level. Figure 1 displays each participant’s measured TEE alongside predicted TEE values based on the DRI (with estimated and inactive PAL) and 25–30 kcal/kg estimates. The 25 kcal/kg estimate underestimated measured TEE in 13 (65%) and overestimated it in 3 (15%) participants. The 30 kcal/kg estimate underestimated TEE in 7 (35%) and overestimated it in 6 participants (30%). The DRI with an inactive PAL underestimated TEE in 9 (45%) and overestimated it in 2 participants (10%). The DRI with an estimated PAL showed the least prediction error, underestimating TEE in *n* = 2 (10%) and overestimating it in *n* = 7 (35%) participants. Among the 4 prediction methods, the 30 kcal/kg was the most accurate to estimate TEE at the group level, showing the lowest bias; however, it fell within ±10% of DLW-measured TEE for only 35% of participants (Figure 2). In terms of individual-level accuracy, the 30 kcal/kg/d estimates had the widest limits of agreement, showing the poorest agreement at the individual level (range: −41.5 to 49.2% or −1030 to 1045 kcal/d) (Table 2).

**TABLE 2:** Total energy expenditure of survivors of colorectal cancer (*n* = 20) measured by double-labeled water in comparison with that predicted by 4 methods, including bias, and limits of agreement^1^.

	TEE (kcal/d), mean ± SD	Bias (kcal/d), mean ± SD	Bias (%), mean ± SD	Proportional bias^2^		Limits of agreement (kcal/d)	Limits of agreement (%)	Absolute limits of agreement (kcal/d)	Absolute limits of agreement (%)
				*r*	*P*				
Measured TEE									
DLW, kcal/d	2467 ± 684								
Predicted TEE^3^									
25 kcal/kg	2062 ± 433^4^	−405 ± 523	−13.5 ± 19.3	−0.525	0.017	−1430, 621	−51.3, 24.3	2051	75.6
30 kcal/kg	2475 ± 520	8 ± 529	3.8 ± 23.1	−0.341	0.141	−1030, 1045	−41.5, 49.2	2075	90.7
DRI (estimated PAL)	2498 ± 410	31 ± 364	4.5 ± 14.4	−0.771	<0.001	−682, 745	−23.7, 32.6	1427	56.3
DRI (inactive PAL)	2232 ± 351^4^	−235 ± 463	−6.1 ± 16.2	−0.756	<0.001	−1143, 674	−37.8, 25.6	1817	63.3

Abbreviations: DLW, double labeled water; DRI, Dietary Reference Intake; PAL, physical activity level; SD, standard deviation; TEE, total energy expenditure.

1 PAL was estimated with International Physical Activity Questionnaire categories converted into its corresponding activity factor according to the Dietary Reference Intake or PAL was equivalent to the “inactive” Dietary Reference Intake equation.

2 Proportional bias determined as Pearson correlation between bias and mean of measured and predicted TEE.

3 All differences (*P* values) between predicted and measured TEE using paired samples *t* test as normality was found with the Shapiro-Wilk test.

4 *P* < 0.05, measured compared with predicted total energy expenditure.

All REE equations, except for Huang and Korth (weight only), showed no significant sex differences in mean bias; thus, males and females were analyzed together. Mean measured REE was 1697 ± 331 kcal/d (range: 1182–2386 kcal/d). Nineteen equations (86.4%) significantly underestimated REE, with the exception of the 21 kcal/kg/d estimate and the Huang and Korth (weight) equations. None of the equations overestimated REE (Table 3). Seven REE equations (31.8%) had a bias within 10% of the REE measured by the WRIC. The smallest (i.e., most accurate) limits of agreement were observed for the Johnstone (−22.3 to 0.6% or −425 to 38 kcal/d) and the Harris-Benedict equations (−21.1 to 8% or −387 to 144 kcal/d). Conversely, the widest (i.e., less accurate) limits of agreement were observed with the Souza-Singer equation (−55.9 to 5.7% or −1096 to 172 kcal/d), which was the only cancer-specific equation included in this study. The proportion of cases in which each equation predicted REE within 10% of the WRIC-measured values is shown in Figure 3. At the individual level, the most accurate equations for predicting REE within 10% (i.e., between 90% and 110%) of measured REE were Harris-Benedict and Müller (65% each), followed by Henry (weight only), Huang (weight only), and Korth (2-compartment body composition equation), at 60% each. Most of the remaining equations predicted REE within 10% of the measured value in ∼50% of participants. Notably, equations that incorporated body composition were not more accurate than those that exclusively included anthropometric measures. For example, the 3 most inaccurate equations in this study incorporated body composition data (i.e., Owen, followed by Mifflin-St. Jeor and Souza-Singer). It is important to note that body composition techniques used to develop these equations varied across studies and included methods such as bioelectrical impedance analysis, DXA, air displacement plethysmography (BOD-POD, COSMED, Concord, CA, USA), and multicomponent models.

**TABLE 3:** Resting energy expenditure of *n* = 20 survivors of colorectal cancer measured by a whole-room indirect calorimeter compared with that predicted by *n* = 22 equations with and without body composition data, including their bias, and limits of agreement.^1^

	REE (kcal/d), mean ± SD	Bias (%), mean ± SD	Proportional bias^2^		Limits of agreement (%)	Absolute limits of agreement (%)
			r	P		
Measured REE						
WRIC (kcal/d)	1697 ± 332					
Predicted REE—Equations without BC^2^						
Harris-Benedict	1575 ± 268^3^	−6.5 ± 7.4	−0.475	0.034	−21.1, 8.0	29.1
Mifflin-St. Jeor	1505 ± 261^3^	−10.7 ± 8.5	−0.459	0.042	−27.4, 6.0	33.4
Owen	1562 ± 261^3^	−7.0 ± 10.9	−0.366	0.112	−28.2, 14.3	42.6
21 (kcal/kg/d)	1732 ± 364	2.2 ± 11.0	0.184	0.437	−19.3, 23.7	42.9
Schofield, weight	1545 ± 233^3^	−8.0 ± 8.5	−0.607	0.005	−24.7, 8.7	33.4
Schofield, height and weight	1566 ± 234^3^	−6.7 ± 9.4	−0.578	0.008	−25.1, 11.8	36.8
Henry, weight	1559 ± 266^3^	−7.5 ± 7.8	−0.448	0.047	−22.8, 7.8	30.6
Henry, height and weight	1558 ± 249^3^	−7.3 ± 8.2	−0.539	0.014	−23.4, 8.7	32.1
Müller	1579 ± 310^3^	−6.6 ± 12.1	−0.119	0.616	−30.4, 17.2	47.6
Huang	1615 ± 282	−4.1 ± 10.3	−0.284	0.224	−24.2, 16.0	40.2
Korth	1677 ± 313	−0.6 ± 11.5	−0.104	0.664	−23.0, 21.9	44.9
Predicted REE—Equations with BC^2^						
Müller, BC	1537 ± 241^3^	−8.5 ± 8.7	−0.559	0.01	−25.6, 8.6	34.2
Korth, 2C	1591 ± 252^3^	−5.3 ± 9.1	−0.528	0.017	−23.2, 12.6	35.8
Korth, 4C	1587 ± 256^3^	−5.5 ± 9.1	−0.505	0.023	−23.3, 12.2	35.5
Wang	1478 ± 215^3^	−11.8 ± 8.7	−0.703	<0.001	−28.9, 5.2	34.2
Cunningham	1445 ± 216^3^	−13.8 ± 8.4	−0.7	<0.001	−30.4, 2.7	33.1
Souza-Singer	1235 ± 166^3^	−25.1 ± 15.7	−0.615	0.004	−55.9, 5.7	61.6
Huang, BC	1520 ± 268^3^	−9.8 ± 8.6	−0.409	0.073	−26.7, 7.0	33.6
Johnstone	1504 ± 258^3^	−10.9 ± 5.8	−0.632	0.003	−22.3, 0.6	22.9
Mifflin-St. Jeor, BC	1394 ± 197^3^	−16.8 ± 8.3	−0.771	<0.001	−33.1, −0.5	32.6
Owen, BC	1366 ± 244^3^	−18.8 ± 8.7	−0.519	0.019	−35.9, −1.8	34.1
Aggregate	1529 ± 242^3^	−9.1 ± 7.8	−0.603	0.005	−24.4, 6.2	30.6

Abbreviations: BC, body composition; REE, resting energy expenditure; SD, standard deviation; WRIC, whole-room indirect calorimeter; 2C, equation using 2-compartment model; 4C, equation using 4-compartment model. Aggregate = mean resting energy expenditure from all predictive equations (without and with body composition data).

1 Proportional bias determined as Pearson correlation between bias and mean of measured and predicted REE

2 All differences (*P* values) between predicted REE and measured REE using paired samples *t* test as normality was found with the Shapiro-Wilk test.

3 *P* < 0.05, measured compared with predicted resting energy expenditure.

Body composition differed between males and females (data not shown); thus, analyses were stratified by sex. Moderate positive correlations were observed between percentage bias from 25 and 30 kcal/kg estimates with BMI and FM, as well as a strong positive correlation with the FM:FFM ratio and a strong negative correlation with ALST/weight (%) (Figure 4). Conversely, percentage bias from the 2 DRI equations (with both estimated and inactive PAL) showed moderate and positive correlations with FFM, FFMI, and ALSTI. A moderate positive correlation with ALST was observed only for the DRI with estimated PAL. In contrast, the percentage bias from the 2 most accurate REE equations (i.e., Johnstone and Harris-Benedict) did not show significant correlations with age, weight, BMI, or body composition.

## Discussion

To our knowledge, this is the first study in a posttreatment CRCS cohort to use 2 state-of-the-art techniques to characterize TEE and REE (i.e., DLW and WRIC, respectively) and the first to assess the accuracy of commonly used predictive equations against these reference methods. Our findings showed that energy requirements varied widely among CRCSs, with standard TEE and REE estimates frequently underestimating true requirements at both group and individual levels. This underestimation indicates that many CRCSs may have higher energy needs than anticipated, potentially leading to inadequate nutritional strategies. Bias in TEE estimates was positively associated with BMI and body composition, and incorporation of a brief PAL questionnaire modestly improved accuracy. Our findings provide new insights into the energy expenditure profile of CRCSs and underscore the need for personalized assessment and nutrition care after cancer treatment.

Gold standard techniques are essential for accurately evaluating newly developed methods and existing ones that have not been previously validated [33]. For instance, although other indirect calorimeters are frequently used as reference methods for assessing REE because of their greater availability, WRICs remain the gold standard because of their strong agreement with the DLW method, showing a mean difference of only 1.3% ± 8.9% between the 2 methods [34]. In contrast, devices such as metabolic carts are ∼10% less accurate than WRICs for REE assessment [35,36], making WRICs the preferred reference method for validation to ensure precise measurements under controlled conditions before clinical application.

To our knowledge, only 3 studies measured free-living TEE in cancer survivors after treatment using the DLW method [[37], [38], [39]]. However, none focused on characterizing TEE, used a reference method such as the WRIC for REE, evaluated the accuracy of predictive equations against measured TEE or REE, or included CRCSs. Carter et al. [1] used DLW to estimate activity energy expenditure in relation to cardiorespiratory fitness and gut microbiota diversity in survivors of breast cancer. Although they reported a mean TEE (∼2010 kcal/d, *n* = 32) lower than that in our study, they did not characterize energy expenditure or explore equation accuracy [37]. Similarly, Copland et al. [38] validated an activity monitor and questionnaire for estimating energy expenditure in survivors of gastric carcinoma [38]. The authors reported a mean TEE (2430 ± 540 kcal/d) that was only ∼30 kcal/d lower than ours. Although questionnaire estimates were compared with DLW-TEE in their study, PAL was calculated using REE from the Deltatrac (Datex) indirect calorimeter, whereas our study employed a WRIC, which closely aligns with DLW (1.3% ± 8.9% overestimation) [34]. Importantly, no attempt was made to characterize TEE or REE or to compare measured and estimated values. Ness et al. [39] used DLW to study energy balance in adult survivors of childhood acute lymphoblastic leukemia but did not examine energy requirement profiles or compare measured and predicted TEE [39]. In their study, energy expenditure values were adjusted by body weight and lean mass, precluding direct comparison with our results. Notably, adjusting TEE (or REE) by dividing it by lean mass (i.e., using a ratio) is inappropriate, as it oversimplifies relationships between energy expenditure and body composition [29,40]. This approach overlooks tissue-specific metabolic variability, leading to biased estimates of energy expenditure as body composition changes. For instance, as body weight increases, the proportion of low-metabolic-rate tissues within lean mass and FFM (e.g., bone and adipose tissue) tends to increase, whereas the proportion of high-metabolic-rate organs (e.g., brain, heart, and liver) tends to decrease, leading to a biased lower estimate of energy expenditure [29]. The alternative is to apply log-log regression models [41]. Thus, our study provides novel, previously unreported insights to the literature, advancing the current understanding in the field.

Although the DRI equation with estimated PAL was the most accurate TEE predictor at the individual level, it under- or overestimated requirements for nearly half the participants. Conversely, although the 30 kcal/kg estimate was the most accurate at the group level, showing the lowest bias, it was accurate for fewer than half of the individuals. The Johnstone and Harris-Benedict were the 2 most accurate REE predictors showing the lowest individual variability, whereas the Souza-Singer equation, the only cancer-specific equation assessed, was the least accurate at the individual level. Notably, the Souza-Singer equation was developed for patients with head and neck cancer, and our findings suggested that cancer-specific equations may need to be tailored to individual cancer types, as equations developed for one type may not generalize to others. Additionally, differences in BMI classifications between our cohort and the original Souza-Singer sample may have influenced the results, as the Souza-Singer equation was developed using patients with malnutrition, whereas most participants in our study had overweight or obesity based on BMI [42]. At the individual level, most REE equations were accurate for no more than half the participants, and incorporating body composition did not improve performance. Cancer and its treatments (e.g., chemotherapy, radiotherapy, and hormonal therapies) can induce long-term physiologic changes in body composition, such as reduced FFM quality and altered fat distribution [29,43]. These alterations may not be fully captured by predictive equations developed in healthy populations, contributing to discrepancies between predicted and measured REE [11,43]. This may help explain why equations incorporating body composition data performed less accurately than those based solely on anthropometric measures. Additionally, body composition techniques varied across studies, whereas we used DXA, which may also have influenced our findings because of differences in precision rates among techniques. However, it is important to highlight that DXA was the technique that showed the strongest agreement with the gold standard multicompartments model [44].

Although predictive equations are convenient for clinical practice, previous studies showed their inaccuracy in estimating energy requirements. For instance, our group previously found several REE equations to be inaccurate compared with metabolic measurements in patients with cancer, including CRC [11]. In another study, we observed similar inaccuracies in patients with active or previous breast cancer, with the Korth and Livingston-Kohlstadt equations emerging as the most accurate among those evaluated [10]. Other studies across various cancer types also reported poor predictive accuracy for individual REE assessment with different equations [12,27,45]. Our findings align with previous research demonstrating that TEE and REE equations often lack accuracy beyond populations with cancer [[46], [47], [48], [49]]. These inaccuracies may stem from the original development of equations in narrow cohorts (e.g., healthy individuals), with limited sample sizes and outdated population characteristics, particularly regarding body weight and composition. Further, differences in equation development methods, individual variability in measured compared with predicted energy expenditure, and inherent estimation error may all contribute to the observed inaccuracies.

In this study, even the most accurate TEE equation at the individual level (i.e., DRI with estimated PAL) led to under- or overestimations of ∼≤1420 kcal/d. Estimating PAL with a questionnaire modestly improved prediction accuracy compared with assuming an “inactive” PAL or using the 25–30 kcal/kg estimates (which do not account for PAL). PALs vary widely among CRCSs during and after treatment, with many being active or highly active [16,50]. Although recently updated DRI equations incorporated various methods for determining PAL [25], they can still lead to misclassification of true PAL [51]. This variability was evident in our study, where measured PAL averaged 1.45 ± 0.23, ranging from 0.97 to 1.83. These findings suggested that using a questionnaire to assess PAL may improve energy requirements estimates in CRCSs. However, wide limits of agreement persisted even when PAL was estimated via questionnaire, likely in part because of inaccuracies inherent in self-reported physical activity data [52]. Similarly, the most accurate REE equation at the individual level (i.e., Johnstone) showed wide limits of agreement, with errors ∼≤460 kcal/d. In clinical practice, such variability in predictive equations could lead to inappropriate energy intake recommendations, causing unintended changes in body weight and composition and resulting in unfavorable health outcomes. Therefore, access to accurate and practical assessment methods is essential to minimize estimation errors and guide appropriate nutritional care. Moreover, we did not apply injury factors to REE equations as this approach seems to overestimate REE. Considering that equations mostly underestimated energy requirements and that applying standard injury factors tends to overestimate the needs of ambulatory patients [27], we hypothesize that using individualized injury factors might improve REE estimates in CRCSs. However, it is important to consider that the application of an injury factor may not be warranted for posttreatment CRCSs. In such cases, applying an activity factor may be more appropriate, as supported by our findings.

All participants in the current study underwent surgery as part of their cancer treatment. Although it is reasonable to expect that energy expenditure increases after surgery, a study [53] involving patients with esophageal cancer showed that REE, measured by indirect calorimetry, was higher in patients before surgery than in healthy controls. REE increased 7 d after surgery but returned to preoperative levels by 14 d after surgery. Another study [54] involving patients with gastric and CRC showed that the REE measured before surgery (∼1370 ± 230) was significantly lower than REE measured ∼1 wk after surgery (∼1470 ± 230, *P* < 0.001). This increase in REE after surgery could be partially explained by elevated body temperature related to postsurgery complications, which led to a 10% increase in REE, as those with uncomplicated surgery experienced only 3% increase in REE ∼1 wk after surgery. Thus, these findings suggest that the impact of surgery on energy expenditure may be lower and shorter lasting than commonly expected. Regarding chemotherapy, this type of treatment seems to decrease REE initially; however, REE seems to increase in the long term after chemotherapy completion, following a U-shaped curve pattern [43]. Because participants in the current study completed treatment for CRC >1 y (median 396 d) before assessments, it is unlikely that surgery or any additional cancer treatment influenced our findings. However, it is important to note that late effects of cancer treatment may still occur, potentially affecting body composition and energy expenditure. For example, chemotherapy has been shown to impair long-term skeletal muscle growth, leading to muscle dysfunction even years after treatment completion [55]. Treatment-induced body composition changes may also include fat infiltration into muscle and chronic inflammation [56], both of which could influence energy expenditure [29]. In addition, CRC surgery can directly reduce muscle mass [57], which may in turn affect energy expenditure [29]. These factors should therefore be considered in future research to clarify their contributions to alterations in body composition and energy expenditure during cancer survivorship.

TEE equation bias was frequently correlated with BMI and body composition, with fat particularly correlated to the 25–30 kcal/kg estimates, whereas muscle (or its corresponding compartments) correlated to the DRI (with estimated and inactive PAL). This suggests that bias may be more pronounced in individuals at the extremes of the BMI spectrum, potentially leading to greater under- or overestimation of energy requirements. This issue is especially relevant for CRCSs, as most participants in our study had overweight or obesity at the time of assessments. Personalized assessments would therefore be especially valuable for those most likely to benefit from tailored interventions, such as those requiring weight loss or gain (e.g., obesity and malnutrition), as assessing all individuals is not currently feasible. This approach could support achieving body weight and composition linked to improved outcomes for patients with or surviving cancer.

When methods to assess energy expenditure are not available in practice, the use of predictive equations or kcal/kg estimates are recommended [13], with regular re-evaluation and monitoring to adjust prescriptions as needed [58]. We recommend selecting the most accurate available equation, based on the best evidence for the specific cancer type, while recognizing its limitations and interpreting results with caution. For example, we found that the Mifflin-St. Jeor equation performed better in patients with different cancer types [11], whereas the Korth and the Livingston-Kohlstadt equations were more accurate in patients with and survivors of breast cancer [10]. In the present study, we found the Johnstone equation to be more accurate for predicting REE and the DRI with estimated PAL for predicting TEE in CRCSs. These require validation in larger cohorts. Future research should explore whether adding relevant cancer-related factors (e.g., cancer type, stage, treatment history and late effects, inflammatory status) [29] to typical predictors of age, weight, height, body composition, and sex would improve equation accuracy. In parallel, efforts should continue to advance bedside technologies for measuring REE. Development of newer portable devices represents a promising step toward broader clinical implementation [59], provided their accuracy in different populations is confirmed. If validated, such tools could support healthcare providers in delivering individualized recommendations. Additionally, multicenter studies are essential to provide large, diverse datasets for developing cancer-specific predictive equations and validating lower-cost methods.

Our sample, composed of posttreatment CRCSs with balanced sex distribution and comparable age and BMI, reflects a large segment of this population [5] and shares metabolic and cardiovascular disease risk profiles with individuals with other cancers, such as breast and prostate [5]. Although certain subgroups (e.g., younger individuals and markedly different body compositions) were underrepresented, physiological links between cancer, treatment effects, and altered energy metabolism are common to multiple cancer types [29]. The combination of high-precision laboratory-based REE measurement (i.e., WRIC) and gold standard, free-living TEE assessment (i.e., DLW) strengthens the applicability of our findings by capturing both the energy used to sustain vital body functions at rest and expended during habitual daily activities. The observed accuracy and bias patterns yielded by equations herein are likely relevant to other cancer survivor groups with similar clinical and functional characteristics, particularly those with obesity-related comorbidities or elevated cardiovascular disease risk [5].

A key strength of our study is the first concurrent use, to our knowledge, of 2 accurate methods, DLW and WRIC [14,15], to measure TEE and REE in a posttreatment CRCS cohort. The inclusion of multiple predictive equations also enhances clinical relevance by evaluating those commonly used in practice. A limitation is the focus on stages II and III cancer survivors, as energy expenditure may differ in those with advanced disease or metastases [29]. Although we achieved our target sample size, larger cohorts may be needed to better assess prediction accuracy in specific subgroups. Thus, the evidence presented in this study should be interpreted with caution.

In conclusion, cancer survivors may have distinct energy expenditure patterns that are not captured by standard predictive equations. Equations commonly used in clinical practice were inaccurate for estimating energy requirements in posttreatment CRCSs at both the group and individual levels. Bias in TEE equations was mostly positively associated with BMI and body composition; however, incorporating a brief PAL questionnaire modestly improved their accuracy. Validating equations in populations with cancer may improve their accuracy. These findings underscore the need for tailored approaches in survivorship care.

## Author contributions

The authors’ responsibilities were as follows – CMP: designed research (project conception, development of overall research plan, and budget acquisition); JFM, CMP: study oversight; APP, JFM, SAP, IMS, HC, RJES, TP, PS, MBS, RE, PJW, CMP: data analysis/interpretation; APP: wrote the first draft of the paper; CMP: acquired funding and had primary responsibility for final content; and all authors: read and approved the final manuscript.

## Data availability

Data described in the article will be made available upon request.

## Funding

This work was supported by Alberta Health Services Cancer Strategic Clinical NetworksTM/Cancer Control Alberta Seed Grant and the Canadian Institutes of Health Research (CIHR) (grant number FRN: PJT-159537), and the Canadian Foundation for Innovation (John R. Evans Leaders Fund, grant number: 34115). APP is supported by the Izaak Walton Killam Memorial Scholarship and has helda graduate studentship funded by the Alberta Women’s Health Foundation through the Women and Children’s Health Research Institute. IMS was supported by Coordenação de Aperfeiçoamento de Pessoal de Nível Superior (CAPES), Brazil, and by a fellowship from Alberta Innovates. CMP is partially supported by Canada’s Research Chair Program. The funders had no role in study design, data collection, analysis, interpretation, or writing of the report, and no role in the decision to submit for publication; the funder imposed no restrictions on publication.

## Conflict of interest

RJES reports receiving consulting fees from Faraday Pharmaceuticals, Actimed Therapeutics, and Helsinn Healthcare and advisory roles for Pfizer and Abbott Nutrition. PS reports receiving grants from Baxter, Nestlé, Fresenius Kabi, and ART MEDICAL and honoraria from Nestlé and Nutricia. MBS reports serving on the advisory board for Viatris. CMP reports receiving honoraria from and/or paid consultancy for Abbott Nutrition, Nutricia, Nestle Health Science, and Novo Nordisk. All other authors report no conflicts of interest.
